# Systems-Level Mapping of the Tumor Microenvironment Reveals Immune-Mediated Mechanisms and Potential Targets in Platinum-Resistant Ovarian Cancer

**DOI:** 10.21203/rs.3.rs-10385637/v1

**Published:** 2026-07-22

**Authors:** Adriana Del Pino Herrera, Miguel A. Martinez, Monica Kim, Kadin El-Bakkouri, David A. Iglesias, Meghan C. Ferrall-Fairbanks

**Affiliations:** 1 J. Crayton Pruitt Family Department of Biomedical Engineering, Herbert Wertheim College of Engineering, University of Florida, Gainesville, FL 32611, Unites States; 2 Department of Computer & Information Science & Engineering, University of Florida, Gainesville, FL 32611, United States; 3 Division of Gynecologic Oncology, Department of Obstetrics & Gynecology, College of Medicine, University of Florida, Gainesville, FL 32611, United States; 4 University of Florida Health Cancer Institute, University of Florida, Gainesville, FL 32611, United States

**Keywords:** gynecologic cancer, treatment resistance, systems biology, multi-modal data, computational deconvolution, gene signature, immune-stromal cross-talk

## Abstract

**Background:**

Ovarian cancer remains the most lethal gynecologic cancer, with limited improvements in patient survival despite targeted therapies and a high recurrence rate (~80%). Current standard-of-care for frontline treatment involves platinum-based chemotherapy, but the emergence of resistant clones limits long-term efficacy. Existing models often overlook critical interactions between cancer cells and their microenvironment. Therefore, we investigated the ovarian cancer microenvironment to identify cell populations and markers driving treatment resistance.

**Methods:**

We employed a multi-modal systems biology approach, integrating multiplex immunohistochemistry, bulk, and single-cell RNA sequencing to characterize the ovarian cancer microenvironment. Cell type composition was quantified using ImageJ (mIF) and computational deconvolution tools (CIBERSORTx, singleR) for benign (n=6–13) and cancer (n=7–20) samples. Platinum-sensitivity was determined by mapping single-cell data to a clinically annotated reference. Differential expression analysis and pathway enrichment were performed to identify key biological processes between benign vs cancer and sensitive vs resistant phenotypes. Additionally, a combinatorial marker identification tool (COMET) was used to determine a resistant signature in the sc-RNAseq dataset, which was validated using pseudotime in sc-RNAseq and the TCGA-OV bulk-RNAseq cohort.

**Results:**

Across modalities, results showed an increase in macrophage and T cell marker expression with an upregulation of inflammatory and immune pathways, alongside decreased fibroblast abundance, in cancer compared to benign tissues. Resistant samples also showed high expression of macrophage and fibroblast markers paired with an enrichment of the epithelial-to-mesenchymal transition pathway while sensitive samples showed high expression of T and NK cell markers and the upregulation of immune pathways. COMET identified two distinct resistant programs: an EMT-associated fibroblast signature characterized by INHBA, TIMP3 and NNMT; and a canonical epithelial ovarian cancer signature characterized by SLPI, MMP7, and WFDC2. Resistant signature scoring of bulk data from the TCGA-OV cohort predicted shorter treatment-free intervals for patients with higher signature scores and longer treatment-free intervals for patients with lower scores.

**Conclusions:**

These findings highlight the importance of tumor microenvironment components, particularly macrophages and fibroblasts, as key contributors to resistance in ovarian cancer and establish a potential resistant signature for biomarker discovery.

## BACKGROUND

Ovarian cancer remains the most lethal gynecological cancer accounting for 20,890 new cases and 12,730 deaths in 2025 (1). Due to the non-specific symptoms of the disease, 80% of patients are diagnosed with advanced stages with a 5-year overall survival of less than 40% (2,3). This leads to the death of 1 in 6 ovarian cancer patients withing the first 3 months of diagnosis (4). High-grade serous ovarian cancer (HGSOC) is the most common subtype accounting for 70–85% of ovarian cancer diagnoses (5). At advanced stages, treatment involves the combination of platinum-based chemotherapy and surgical resection (6). Following frontline platinum-based chemotherapy and surgery, maintenance therapy with poly ADP-ribose polymerase (PARP) inhibitors, is considered in certain populations. While initial response to these treatments is often favorable, up to 80% of ovarian cancer patients recur within 2 years (7). Once a patient develops resistance to first-line therapies, there are fewer therapeutic options to prolong the lifespan of these patients.

Despite the development and use of novel targeted therapies, these therapies have not been accompanied by improved outcomes for the majority of ovarian cancer patients and recurrence remains the main obstacle for treatment efficacy. Ovarian cancer is a heterogenous disease with different histological, molecular, and epigenetic profiles. Known mechanisms of chemotherapy resistance include abnormal membrane transport, alterations of DNA damage repair maintaining cell viability, and multidrug resistance (8), which increases treatment complexity and limits single molecule targets in ovarian cancer patients (9). Immunotherapies have also been explored for the treatment of this lethal disease with variable results and not enough positive progress in clinical trials (10) to move the needle in frontline treatment. These challenges highlight the key gap in understanding targetable mechanism(s) that drive disease progression and platinum-resistance in ovarian cancer.

The tumor microenvironment (TME) plays a crucial role in ovarian cancer progression. It is characterized by an acidic pH, low oxygen and nutrient availability, and recruitment of supporting cells to contribute to immunosuppression, cancer progression and resistance (11). Cells such as macrophages, T-cells, and fibroblasts and extracellular matrix proteins contribute to tumorigenesis (12,13). Both fibroblast and macrophages alter their phenotypic expression when near ovarian cancer cells producing cancer associated fibroblasts (CAFs) and tumor associated macrophages (TAMs), respectively (14). CAFs are induced by the inflammatory and hypoxic conditions of the TME, with a pro-tumorigenic phenotype and promoting resistance to chemotherapies (15). Similarly, the hypoxic TME also induces TAMs recruitment. Whereas, TAMs can present a hybrid M1-like (pro-inflammatory) and M2-like (anti-inflammatory) phenotype, often M2-like macrophages dominate in the ovarian cancer TME (11,16). These M2-like TAMs will reduce immune cell recruitment and action, promoting therapy resistance.

**Figure 1.**
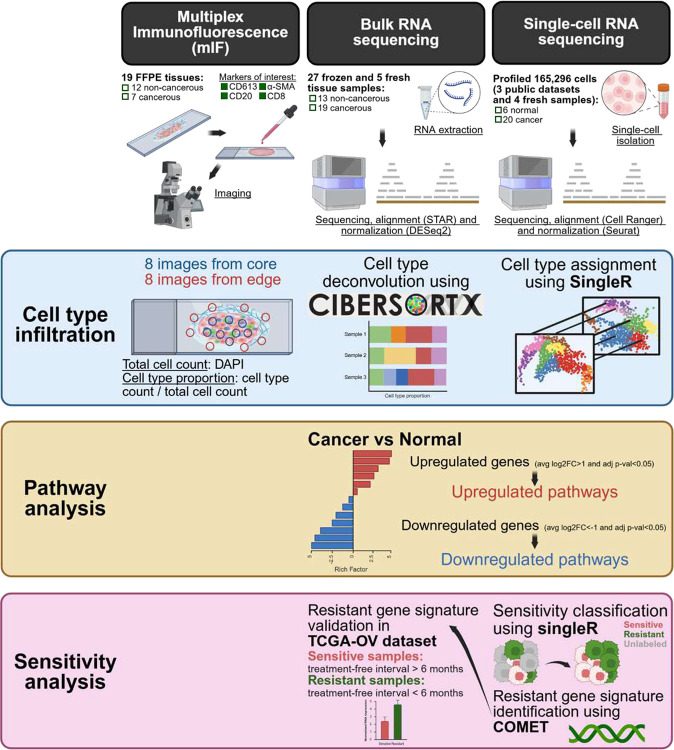
Schematic of methodology for the study of the tumor microenvironment in ovarian cancer.

Here, we focused on a systems-level approach combining protein and transcriptomic information to unravel mechanisms of ovarian cancer disease progression compared to normal ovarian tissues as well as resistance mechanisms (Figure 1). Three technologies were explored: multiplex immunofluorescence (mIF) to unravel differences in protein expression and bulk RNA- and single-cell RNA-sequencing (bulk RNA-seq and scRNA-seq) to unravel differences in transcriptomic information about cellular phenotypes at play. These tools identified key immune and stromal components in tisues from benign/normal ovary and ovarian cancer as well as differences in upregulated and downregulated pathways. The agreement between the modalities was assessed suggesting the need for one modality or another to unravel certain disease mechanisms. Additionally, a variety of samples were compiled both from publicly available sources and from specimens processed in-house increasing the sample size and heterogeneity of the dataset for more reliable results. Lastly, platinum-sensitivity was assigned to all the cells in the scRNA-seq cohort providing not only characteristic resistant cell types but also resistance signatures that could improve treatment strategies.

## METHODS

### Multiplex Immunofluorescence Cell Quantification

Formalin-Fixed Paraffin-Embedded (FFPE) ovarian cancerous, benign and normal ovary tissues were collected from the UF Clinical and Translational Science Institute (CTSI) Biorepository (BioR) in accordance to approved IRB protocol IRB202200692. Samples were sectioned and stained by the UF Molecular Pathology Core to identify different tumor cells using multiplex immunofluorescence (mIF): M2 macrophages (CD163; Abcam #abm87099), B cells (CD20; Proteintech #6027 1-1-Ig), T cells (CD8; Novus Biologicals NBP2-54595AF627), and fibroblasts (α-SMA; Invitrogen #53976082). Additionally, the nuclei for all the samples was labeled with a DAPI stain.

Entire tissue slices were imaged using the ECHO Revolution microscope at a 10x magnitude. Eight core and eight edge tissue tile images were selected at random for quantification. Once the images were selected, ImageJ was used to perform cellular quantification by using a threshold to capture the highest intensity objects to then segment them (see **Supplemental Methods** for more details). The samples were then grouped into non-cancerous (n = 12 samples, containing normal and benign samples) or cancerous (n = 7 samples) (metadata available in **Supplemental Table S1**). For each sample, cell proportions were collected for core and edge areas, which were scaled for plotting. Cell type proportions across the different groups were compared using pairwise non-parametric t-tests.

### Fresh Sample Cell Dissociation

Five fresh ovarian cancer patient samples were collected in collaboration with the University of Florida Department of Obstetrics and Gynecology in accordance to approved IRB protocol IRB202200692. Upon surgical removal from the patient and verified by pathology, tumor samples were preserved for a maximum of 24 hours in MACS tissue storage solution (Miltenyi Biotec #130-100-008). Tumors were then dissociated into single-cell suspensions following the protocol of the tumor dissociation kit (Miltenyi Biotec #130-095-929) and the gentleMACS dissociator following manufacturer’s instructions (more details available in the **Supplemental Methods**). Four samples were later processed for single-cell RNA-seq and all samples were processed for bulk RNA-seq.

### Ovarian Cancer Cell Type Reference

To obtain cell type annotations for ovarian cancer samples, an ovarian cancer reference was generated from two high grade ovarian cancer samples publicly available in Gene Expression Omnibus (GEO) under accession number GSE173682 and the cell type classification previously established by Regner. M et al. (17) (**Supplemental Table S4, Supplemental Figure S3B, Supplemental Methods**).

### Bulk RNA-seq Sample Preparation and Data Preprocessing

Flash frozen ovarian cancerous, benign, and normal ovary samples were also collected from the UF CTSI BioR in accordance to approved IRB protocol IRB202200692. RNA was extracted from 32 samples using the Qiagen RNeasy Mini Kit (Qiagen #74104) following manufacturer’s protocols (more details available in the **Supplemental Methods**). Upon extraction, RNA was quantified using a NanoDrop. Samples with an RNA content between 40–100 ng/μL and an OD 260/230 purification ratio higher than 1.6 were used for sequencing. RNA was sequenced by the University of Florida Gene Expression and Genotyping Core Facility (ICBR; RRID:SCR_019145) at 75 million reads per sample using ribosomal depletion and the Illumina NovaSeq X Series 10B 2x150.

Sequences were provided from the ICBR in compressed FASTQ file format for each sample. STAR (2.7.11b) (18) was used to align samples to the human genome and to output gene counts. The unstranded gene counts for each sample were input to R (4.4) and merged into a singular count matrix. The ENSEMBL genes were converted to gene symbols using mapIds from the org.Hs.ed.db (3.21.0) package. Unmapped genes were deleted from the count matrix and in the case of duplicated gene with the highest expression column were kept in the matrix. Genes were also removed from the matrix if counts per million (CPM) where less than 0.5 and matrix was log-normalized using EdgeR (4.2.2). Samples were grouped into non-cancerous (n = 9 normal ovarian samples and n = 4 benign ovarian samples) and cancerous (n = 19 ovarian cancer samples) groups (metadata for all samples available in **Supplemental Table S2**) and DESeq2 (1.44.0) was used to perform differential gene expression between these two groups. A non-parametric one-tail Student’s t-test was used to compare gene expression values between non-cancerous and cancerous groups.

In order to infer cell type proportions across the different samples, CIBERSORTx (19) was used as a deconvolution tool. The ovarian reference containing characteristic cell types as previously described in the Methods and **Supplemental Methods** was input into CIBERSORTx as the signature matrix file. The raw count matrix for the 32 bulk samples output from STAR was converted into counts per million and uploaded as a mixture file. CIBERSORTx ran a query using the signature matrix and mixture files to output a table including cell type proportions of each samples. Samples were grouped based into non-cancerous and cancerous groups and the cell type proportions were averages per group.

### Single-cell RNA-seq Sample Preparation and Data Preprocessing

Dissociated cell samples were thawed as outlined by 10X Genomics (see **Supplemental Methods** for more details). Cells were then counted manually using a hemocytometer and viability was assessed. Samples with 70% viability were prepared for sequencing and samples with clumped cells and lower viability were ran through the LeviCell system (Levistabio) to remove dead cells and cell clumps. After clean-up, 2,400–9,200 cells were used for sequencing. Sample preparation for single-cell sequencing was done by the ICBR. Samples were sequenced using the 5’ single-cell RNA sequencing 10X Genomics Library Preparation including T and B cells V(D)J libraries. 50,000 reads were collected per cells sequenced with the Illumina NovaSeq X Series 10B 2x150. Sequences were then received from the ICBR in compressed FASTQ formats. Sequences were aligned to the human genome using Cellranger multi 8.0.0 (10X Genomics) (20) and only the gene expression counts were used for further analysis.

In addition to the fresh samples processed and sequenced in-house, three additional datasets were analyzed. GSE154600, GSE181955 and GSE184880 (21–23) were chosen to capture a landscape of both normal ovary and ovarian cancer samples, treated and treatment naïve samples, various staging information, and annotated platinum-sensitivity/response was reported across the samples (metadata compiled in **Supplemental Table S3**). Once compressed FASTQ files were compiled, Cellranger counts 8.0.0 was used to align the samples to the human genome GRCh38. In total, 26 samples were collected, 6 normal ovarian samples and 20 ovarian cancer primary samples, 4 of which were processed from fresh tumors in-house.

Cellranger outputs were input to R (4.4) and converted to Seurat objects (5.3.0) (24) annotated with metadata. Individual objects were merged to a single object with a total of 192,118 cells. Filtering removed cells with RNA features 2 standard deviations higher than the mean and less than 450 features and cells with greater than 25% content resulting in a total number of 165,296 high-quality cells. The object was then normalized (SCTransform, v0.4.2) (25) and dimensionality was reduced (PCA). The object was harmonized to remove batch effects to account for differences in technologies (3’ vs 5’) and processing protocols using Harmony (v1.2.3) (26) (**Supplemental Figure S3C**). Samples were then clustered using graph-based clustering (Louvian algorithm, resolution 0.025) and visualized with UMAP.

SingleR (v.2.11.3) (27) was used to determine individual cell type assignments and used the annotated single-cell reference previously described. Both the SCTransformed reference and the table with annotated metadata were used as inputs for the singleR function and the unassigned cells as the query. For each sample, the proportions for each cell type was calculated and compared between normal samples and ovarian cancer samples. Additionally, dot plots for the expression of canonical markers of different cell types were created to compare the expression levels between normal and cancer cells.

### Single-cell RNA-seq Platinum-Sensitivity Classification

Five ovarian cancer samples from the GSE154600 were clinically identified as platinum-sensitive, platinum-resistant, or refractory. These samples were subset to create a platinum-sensitivity reference. Refractory cells were labeled as resistant creating a group of resistant or non-responder cells. Equal sized groups were used for the sensitive and resistant cells in the reference (see **Supplemental Methods** for details) to predict single-cell sensitivity status of unlabeled cancer cells. Then the expression of canonical cell type markers was assessed between normal, sensitive, and resistant cells. Cell type enrichment per sensitivity and treatment category was also investigated using the cell type assignment already performed using singleR.

### Differential Gene Expression

Differential gene expression was performed using DESeq2 (1.44.0) (28) in bulk RNA-seq samples and using the Seurat function FindMarkers() in single-cell RNA-seq samples. Lists of differentially expressed genes between cancerous and non-cancerous groups for both bulk RNA-seq and single-cell RNA-seq were computed and the significant genes (adjusted p-value < 0.05) were subset. The similarities between the two lists (from bulk and single-cell RNA-seq) of all significant genes, only upregulated (average log2FC > 0) significant genes and only downregulated (average log2FC < 0) significant genes were assessed by calculating the Jaccard index. An index close to 1 indicates complete similarity between the two sets while an index closer to 0 means complete dissimilarity. The matching significant differentially expressed genes obtained from both bulk and single-cell RNA-seq technologies were related to biological processes and plotted using Sankey plots.

Differentially expressed genes were then used for pathway analysis. Both bulk and single-cell RNA-seq gene lists were filtered to only include the most significant genes (average | log2FC | >= 1, adjusted p-value <=0.05). Gene lists were converted to ENTREZIDs using the bitr function from the clusterProfiler (v4.17.0) package (29). Additionally, a gene list including all the genes expressed in the bulk- and sc-RNAseq datasets was curated by converting the gene symbols of to ENTREZIDs and used as the background gene list. The upregulated or downregulated and background gene lists for bulk or single-cell RNA-seq modalities were used as inputs to identify Hallmark pathways from GSEA using the Enricher function (30). The −log10(adjusted p-values) obtained for each pathway and was used to create Sankey plots for changes in pathway expression over disease progression identified in bulk and single-cell RNA-seq.

Differentially expressed genes between resistant cells in single-cell RNA-seq and all the other cells (normal and sensitive cancer cells) were also obtained and separated into upregulated (average log2FC >= 1, adjusted p-value <=0.05) and downregulated significant genes (average log2FC <= −1, adjusted p-value <=0.05). These gene lists were then used to identify Hallmark pathways with altered expression and plotted in a chord plot. Furthermore, to identify putative markers of platinum-resistance, another round of differential gene expression was performed with COMET (31) was used to identify a ranked list markers associated with platinum-resistance with quantified true negative and true positive expression across the single-cell RNA-seq samples. Genes of interest were those ranked as highest enrichment (resulting in a small hgrank value) and highest expression change between resistant and all other cell groups (resulting in a small fcrank value). These values (hgrank and fcrank) were added, and a histogram was used to determine a cut off of the top 15^th^ percentile best scoring genes (**Supplemental Figure S10A**).

### Pseudotime Analysis with Monocle

Monocle3 was used to perform pseudtime analysis using single-cell RNA-seq data. Clusters of interest were subset from the full Seurat object (cluster 2 was analyzed as the epithelial cluster and cluster 3 as the fibroblast cluster). The count matrix, metadata, and gene names were extracted to create a cds object recognizable by Monocle3. UMAP embeddings and clusters identities were added to the cds object. The cds object was pre-processed (preprocess_cds) and clustered (cluster_cells) to obtain monocle clusters. The function learn_graph was used to find the different possible pseudotime trajectories and order_cells was used to identify the particular trajectory of interest picking a resistant cell as the starting point. SLPI and CTHRC1 were selected and their expression across the pseudotime trajectory of interest was visualized using the function plot_genes_in_pseudotime.

### Bulk RNA-seq Validation with TCGA Dataset

Bulk RNA-seq data from ovarian cancer patients was downloaded from the TCGA-OV dataset obtained from The Cancer Genome Atlas (TCGA, https://portal.gdc.cancer.gov/) (32). Patients were stratified based on treatment information availability as explained in the CONSORT diagram in **Supplementary Figure S7A**. Patients with metadata including days to treatment start, days to treatment end, therapeutic agent names, and vital status were kept. Patients were stratified by those that had received a platinum-based therapy as their first treatment versus those that had received a second treatment after. From 608 patients in the TCGA-OV cohort initially, 102 met these criteria and had received a platinum-based chemotherapy as first treatment and a follow up treatment afterwards. The length between the end date from the first treatment to the start date of the second treatment was measured and labeled as treatment-free interval (TFI). Patients with TFIs longer than 180 days (n=43) were classified as sensitive while patients with TFIs shorter than 180 days (n=59) were classified as resistant in alignment with clinical definitions of patients with 6 months of more between treatment regimens can be classified as platinum-sensitive (33).

For these patients, raw transcriptomic information was downloaded using the GDCquery function from the TCGAbinlinks (v2.32.0) package (34), to then create a count matrix in R. ENSEMBL symbols were converted to gene symbols using clusterProfiler (v4.12.6) (29). Genes with less than 0.5 counts per million (CPM) were filtered out and the count matrix was log-normalized using EdgeR (v4.2.2) (35). The normalized expression of different markers between sensitive and resistant groups were plotted using bar plots and compared using non-parametric Students’ t-tests. The function gsva() (v2.4.9) (36) was used to calculate an enrichment score for each TCGA-OV patient based on expression of genes in the COMET resistant signature identified. Survival correlations were created by separating the samples into above and below the median of the COMET signature score. The Kaplan-Meier curve method on GraphPad Prism based on first TFI length and vital status of the patients reported by TCGA was used to compared high and low COMET score groups.

### Mammalian Cell Culturing and Cell Counting

Tyk-nu (sensitive) and Tyk-nu cp.r (cisplatin-resistant) ovarian cancer cells were purchased from the JCBR Cell Bank (37). Cells were cultured EMEM supplemented with 10% fetal bovine serum, 1% penicillin/streptomycin and 1% L-glutamine (200mM). Every other passage, 1μM of cisplatin was added to the Tyk-nu cp.r cells to maintain resistance. For cell counting purposes, sensitive and resistant cells were fluorescently labeled with Incucyte Nuclight Red and Green Lentivirus (4624 and 4625) respectively, using a MOI of 4 and selected with 3 μg/mL of puromycin. Once a fluorescently stable population was created and expanded, co-culture experiments were performed.

Sensitive and resistant cells were passaged and seeded in triplicate in 24-well plates at a concentration of 40,000 cells/mL. Under standard media conditions, cells were allowed to attach, and images were taken every 24 hours, changing the media every 48 hours. Cell counts were abstracted from images using QuPath (see **Supplemental Methods** for more details). Under M2 TAM media conditions, initially THP1 monocytes (ATCC) were seeded in a 100mm dish at a concentration of 100,000 cells/mL in RPMI supplemented with 10% fetal bovine serum, 1% penicillin/streptomycin and 1% L-glutamine (200mm). PMA at concentration of 125ng/mL was added to the dish at the time of seeding to allow cells to attach. After 24 hours, differentiated M0 macrophages were rinsed with 10mL 1xPBS and a 1:1 ratio of complete RPMI:EMEM was added to the dish. After another 24 hours, IL-4 at a concentration of 25 ng/mL and IL-13 at a 25 ng/mL concentration of was added to the dish to differentiate M0 macrophages to M2 macrophages. After 48 hours, media was changed to 1:1 ratio fresh RPMI and cancer cell conditioned EMEM that was collected from 60–90% confluent Tyk-nu and Tyk-nu cp.r flasks, spun down at 3200xg for 10 minutes and filtered with a 0.2um filter. After 48 hours, M2 macrophages had acquired M2 TAM properties and media was collected spun down at 3200xg for 10 minutes. This media was now considered M2 TAM conditioned media. In 24-well plates, co-cultures under M2 TAM conditioned media were seeded and after 24 hours the M2 TAM conditioned media was added to the wells. Then 48 hours after seeding, cells were imaged as described.

### Growth Dynamics and Parameter Estimation

Once the cell counts were collected, they were input into Julia to calculate model parameters. Logistic ordinary differential equation curves, $\frac{dN}{dt}=rN(1-\frac{N}{K})$, were fitted to all datasets, where N(t) is the population size at time t, r is the cell growth rate (in day^−1^), and K is the carrying capacity. Growth rates and carrying capacities were calculated using the DifferentialEquations.jl and DiffEqParamEstim.jl (38). The ODEproblem function was used to define the problem and a loss function was created to minimize the loss between experimental and simulated curves using the Rodas5() solver and a global optimization algorithm in the BlackBoxOptim package (39).

### Statistical Analysis

Unless stated otherwise, all statistical analysis was performed using GraphPad (v10.3.1). Comparisons of gene expression or cell proportions between two groups was performed using non-parametric Student’s t-test. Bland-Altman bias and limits of agreement were computed using the Bland-Altman method comparison in GraphPad. Survival plots were created using the Kaplan-Meier methods in GraphPad.

## RESULTS

### Macrophage and T-cell markers are upregulated in cancer samples while fibroblasts are downregulated compared to normal samples (1215 words)

**Figure 2.**
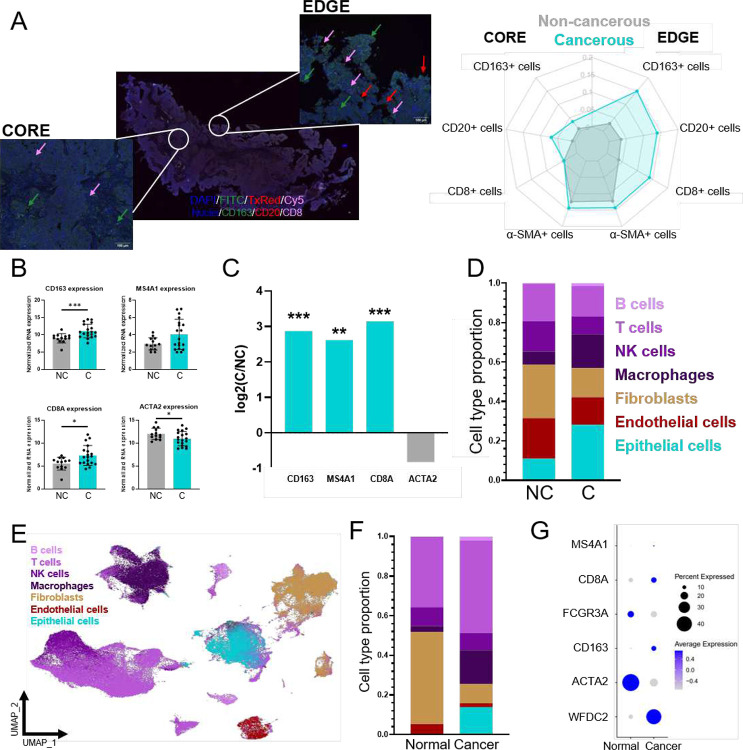
Cancer samples showed an upregulation of macrophages and T-cells, while normal samples showed an upregulation of fibroblast across mIF and bulk and single-cell RNA-seq. **A**. mIF was used to evaluate protein expression of core and edge regions of non-cancerous (n=12) and cancerous (n=7) FFPE samples. Edge regions showed the highest proportions of cells compared to core regions and macrophages, B- and T-cells were highly present in cancerous samples compared to non-cancerous. **B**. The normalized expression of four markers of interest were evaluated across bulk RNA-seq non-cancerous (n=13) and cancerous (n=19) frozen and fresh ovarian samples. Macrophages and T-cells showed higher expression of CD163 and CD8 in cancerous compared to non-cancerous samples, while ACTA2, a fibroblast marker, showed higher expression in non-cancerous compared to cancerous samples. **C**. Differential gene expression between cancerous and non-cancerous samples in bulk RNA-seq. CD163, CD8, and MS4A1 were significantly upregulated in cancerous samples, while ACTA2 was downregulated compared to non-cancerous samples. **D**. Deconvolution of bulk RNA-seq samples using CIBERSORTx and an ovarian cancer reference showed that cancerous samples have a higher proportion of epithelial, B-cells, and macrophages, while non-cancerous samples had a higher proportion of T-cells and fibroblasts. **E**. UMAP showing cell type distribution after running singleR with an ovarian cancer reference. Each cell types falls into a different cluster. **F**. Deconvolution of single-cell RNAseq samples after singleR showed that B-cells, macrophages, T-cells, and epithelial cells had a higher proportion in cancer samples, while fibroblasts have a higher proportion in normal samples. **G**. Dot plot show average expression and percent of cell expressing markers of ovarian cell types in normal versus cancer cells. NK-cells and fibroblasts markers had the highest expression in normal cells, while B-cells, T-cells, macrophage, and epithelial cell markers had the highest expression in cancer cells. * p-value < 0.05; ** p-value < 0.01; *** p-value < 0.001

Three modalities were explored to unravel the phenotypic composition of the ovarian cancer tumor microenvironment. mIF was used to study cellular composition based on protein expression, while bulk and single-cell RNA-seq focused on transcriptomic phenotype refinement at the tissue and single-cell levels, respectively. Protein expression profiles for CD163, CD20, CD8 and α-SMA were evaluated in tissues slices from FFPE blocks from non-cancerous (n = 12 samples) and cancerous (n = 7 samples) ovarian tissue that were stained. Entire slices were imaged, and eight core and eight edge regions of interest (ROIs) were chosen at random to determine cell type proportions of each population of interest relative to the total number of cells. For all immune cell markers (CD163, CD20 and CD8), core areas showed a lower cell type proportion compared to edge areas regardless of cancerous classification (Figure 2A **and Supplemental Figure S1**). CD163+ cells showed higher proportion in cancerous tissue both in core and edge regions compared to non-cancerous tissues (p-value: 0.007 and 0.0435, respectively). In core areas, there is a 3.61-fold change increase in macrophage proportion from non-cancerous to cancerous samples and a 5.49-fold change increase in edge areas. For CD20+ cells, there is a 9.12-fold change increase (p-value: 0.0052) from non-cancerous to cancerous samples in core areas and a 4.45-fold change increase (p-value: 0.0171) in edge areas. CD8+ cells showed significant changes when comparing edge areas which resulted in a 3.75-fold increase (p-value: 0.0221) in CD8+ cell proportion from non-cancerous to cancerous samples. Lastly, the cell type proportions for α-SMA+ cells across samples and regions did not show any significant changes (**Supplemental Figure S1**). mIF was able to discern differences in cell type proportions across cancer and non-cancerous samples.

Frozen (n = 27) and fresh (n = 5) samples from non-cancerous (n = 13) and cancerous (n = 19) ovarian tissues had RNA extracted for total RNA sequencing. After alignment and normalization, RNA expression of canonical markers for different cell types in the ovarian cancer microenvironment were evaluated between non-cancerous and cancerous groups. CD163 and CD8A genes showed higher expression in cancerous samples compared to non-cancerous samples (1.23-fold change, p-value: 0.0007; and 1.32-fold change, p-value: 0.0185 respectively). However, the expression of the ACTA2 (α-SMA) marker showed a decrease of 1.09-folds from cancerous to non-cancerous samples (p-value: 0.0297). While not significant, MS4A1 (CD20) expression increased 1.37-fold change (p-value: 0.057) between non-cancerous and cancerous samples (Figure 2B **and Supplemental Figure S2**). Higher immune-cell infiltration indicated that tumors favor the recruitment of these cell types to inhibit or promote cancer progression. Similarly, differential gene expression between cancerous and non-cancerous samples showed the upregulation of CD163, MS4A1 and CD8A with log2(FC) values of 2.87 (adjusted p-value: 2.65E-6), 2.61 (adjusted p-value: 0.001558) and 3.14 (adjusted p-value: 1.24E-5) respectively in cancerous samples compared to non-cancerous samples. The ACTA2 gene was downregulated in cancerous samples with a log2(FC) of −0.82 (adjusted p-value: 0.147) (Figure 2C).

To deconvolute cell type proportions across all the samples, CIBERSORTx with a curated ovarian cancer reference (**Supplemental Figure S3B**) was used to assign cell type proportions across the 7 types in the reference for the 32 bulk RNA-seq samples. Since ovarian cancer has an epithelial origin, the proportion of epithelial cells tripled from non-cancerous to cancerous (7.43% in non-cancerous and 22.37% in cancerous) as expected. Immune composition showed similar increases, where the proportion of B-cells and macrophages quadrupled and doubled, respectively, between non-cancerous and cancerous tissues. The proportion of T-cells, however, showed an 18% decrease from non-cancerous to cancerous sample, discordant with CD8 RNA expression results (Figure 2D **and Supplemental Figure S2**). The fibroblast proportion was almost half in cancerous tissues compared to non-cancerous tissues, similar to previous results. RNA-seq indicated the favorable recruitment of immune cells to the ovarian cancer tumor microenvironment, while the stromal structure of the tumor site gets lost with the downregulation of fibroblasts.

Lastly, 26 single-cell RNA-seq samples were used to explore transcriptomic differences at a higher resolution. Twenty-two of the samples were from publicly available datasets (21–23) and four samples were processed in house. Six samples were from normal ovarian tissues, while twenty were from cancer tissues (additional metadata available in **Supplemental Table S1**). All samples were aligned, filtered, normalized, and batch corrected before downstream analysis. The samples clustered into 8 different clusters using graph-based clustering. Clusters 1, 2, 4, and 6 are composed of mostly cancer cells (around 90%) while cluster 3 is the only cluster that has a higher proportion of normal cells compared to cancer cells (53% normal and 47% cancer cells) (**Supplemental Figure S3D**).

SingleR was used for cell type assignment based on an annotated ovarian cancer reference. The assignment for each cell type was verified based on the expression of canonical markers (**Supplementary Figure S3E**) previously established. The gene makers CD8A, NCAM1/CD56, CD163, ACTA2/α-SMA, ESM1 and CD19 showed highest expression in cells assigned as T cells, NK cells, macrophages, fibroblasts, endothelial cells and B cells, respectively (40–45). The marker WFDC2/HE4 (46) was used to verify the assignment of epithelial cells, which was highly expressed in both epithelial labeled cells and other epithelial cells which were merged with the epithelial cells. Immune cell types such as B-cells, macrophages, and T-cells showed a higher proportion in cancer samples compared to normal by 26-, 5.8-, and 1.3-fold changes, respectively. Epithelial cells also showed an 85-fold increase between cancer and normal samples. Fibroblasts showed the opposite trend with a higher proportion by 4.8-fold changes in normal samples compared to cancer samples (Figure 2F **and Supplemental Figure S3A**).

As in the other modalities, single-cell expression of CD163, MS4A1, and CD8A markers showed increased expression in cancer cells compared to normal (Figure 2G). ACTA2 expression was reduced in cancer cells compared to normal cells. Across the three modalities, immune markers such as CD163 expression/ macrophage markers, MS4A1 expression / B-cell markers, and CD8A expression / T-cell markers were upregulated in cancer samples compared to normal samples. Particularly macrophages and T-cells showed the highest fold change increases between cancer and normal samples, indicating that these cell types were being recruited to the tumor microenvironment. Fibroblast marker and cell type assignment showed discordance across modalities, but a more definitive trend of depletion in cancerous samples compared to normal tissue.

Furthermore, agreement between the modalities was assessed using Bland-Altman plots to quantify the potential bias given samples that were processed into at least two of the modalities (see **Supplemental Methods** for more details). When comparing the agreement between bulk RNA-seq and mIF, there was the highest agreement for CD163+, CD20+, and α-SMA+ cells and the corresponding RNA-seq expression profiles (**Supplemental Figure S4A**). However, CD8+ cells show higher disagreement between technologies mIF and bulk RNA-seq expression in a density-dependent manner where mIF could detected rare populations than the bulk data (**Supplemental Figure S4A**). Similar comparisons were made between bulk RNA-seq and single-cell RNA-seq (**Supplemental Figure S4B**), which given the small sample size (n=4) did not identify any significant bias due to technology. Additionally, differential gene expression between cancerous and non-cancerous samples was performed for bulk- and single-cell RNA-seq datasets to identify upregulated and downregulated Hallmark pathways. Results showed 11 shared upregulated pathways between modalities (**Supplemental Figure S5**). The most significant were immune and inflammatory pathways such as the tumor necrosis factor-alpha TNF-α signaling via nuclear factor-kB and the inflammatory response pathway (adj. p-value for bulk- and single-cell RNA-seq < 0.0001). The epithelial to mesenchymal transition (EMT) pathway, however, was downregulated in both technologies (adj. p-value < 0.0001).

### Macrophages and fibroblasts play a role in resistance development regardless of treatment status (972 words)

**Figure 3.**
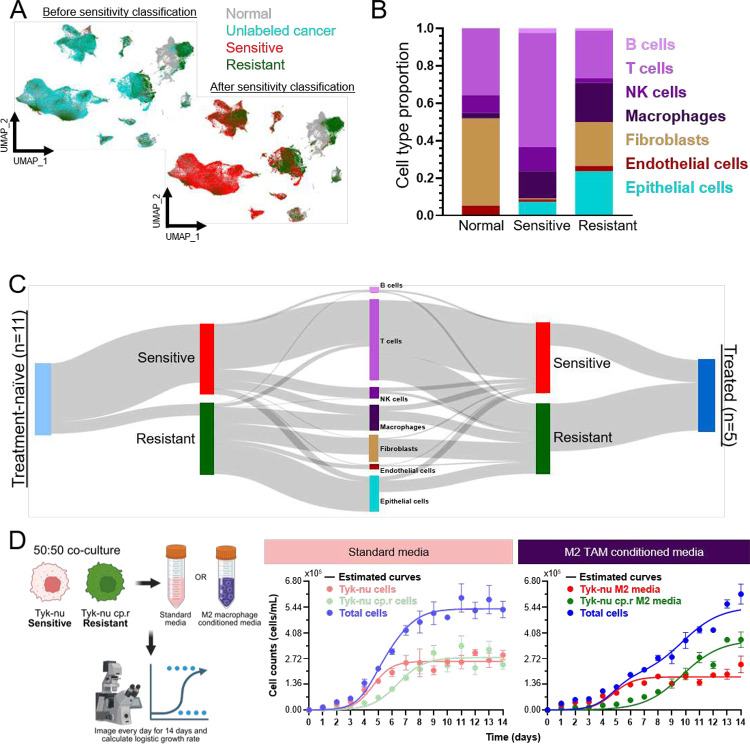
Macrophages and fibroblasts contribute to the development of resistance pre- and post-chemotherapy. **A**. UMAP shows single-cell platinum-sensitivity reference and after classification with singleR. **B**. Single-cell cell type proportions of normal, sensitive and resistant cells. Resistant cells had the highest proportion of macrophages, fibroblasts, and epithelial cells, while sensitive cells had the highest proportion of T-cells. **C**. Sankey plot of treatment-naïve and treated cells classified based on sensitivity and then based on cell type. Both treatment-naïve and treated resistant compartment were dominated by macrophages, fibroblasts, and epithelial cells. **D**. Co-cultures of sensitive and resistant ovarian cancer cells (Tyk-nu and Tyk-nu cp.r) *in vitro* under standard media and M2 tumor-associated macrophage (TAM) conditioned media showed similar total population size at day 14 with resistant cells dominating in the population when exposed to M2 TAM conditioned media.

Since immune and stroma/structural related cells and pathways were dysregulated in ovarian cancer compared to normal tissue, we aimed to identify the role these systems play in platinum-resistance. Of the single-cell RNA-seq cohort, 5 samples were clinically annotated with their platinum-sensitivity/response. SingleR was leveraged to classify each cell as platinum-sensitive or platinum-resistant using a platinum-sensitivity reference as previously described (Figure 3A **and Supplemental Figure S6A**). The cell type classification previously performed was now grouped into normal, sensitive and resistant categories (Figure 3B **and Supplemental Figure S6B-C**). Sensitive samples contained the highest proportions of B-cells, T-cells, and NK-cells compared to normal and resistant samples by 2–32-fold increase in B-cells, 1.7–2.4-fold increase in T-cells, and 1.4–5.7-fold increase in NK-cells. Resistant samples had the highest proportions of macrophages by 1.5–7.2-fold increase compared to sensitive and normal samples. Additionally, the fibroblast proportion was 1.6-fold higher in resistant samples compared to sensitive samples. These trends also aligned with RNA expression profiles of canonical markers for each cell type showing higher CD163 and ACTA2 expression in resistant cells compared to sensitive (**Supplemental Figure S6D**).

This immune infiltration was also validated in a TCGA ovarian cancer cohort with bulk RNA-seq samples and treatment data available (**Supplemental Figure S7C**). TCGA samples were classified as platinum-sensitive if they had a platinum-free interval greater than 180 days from their first platinum-based treatment until the next treatment and resistant if that interval was less than 180 days. However, when the expression of individual B-cell, T-cell, macrophage, and fibroblast markers (MS4A1, CD8A, CD163 and ACTA2, respectively) was compared between these sensitive and resistant groups there were no statistically significant differences (**Supplemental Figure S7B**). This suggests the need for more robust phenotypes derived from the single-cell data to define relevant markers that discern differences in immune infiltration between sensitive and resistant samples.

Of the cohort of 26 single-cell samples, 11 were treatment-naïve and 5 were after treated with chemotherapy (Figure 3C). As expected, treatment drove dominance of resistant cells resulting in treated samples with resistant cell proportions 4.1-fold higher compared to treatment-naïve samples. Sensitive cells in both treatment-naïve and treated groups were dominated by T-cells (61.7% of sensitive cells for treatment-naïve and 77% of sensitive cells for treated) suggesting that an immune response was present even after treatment. In treatment-naïve samples, resistance was largely characterized by epithelial cells, fibroblasts, and macrophages (43%, 23.5%, and 23.7% of treatment naïve-resistant cells, respectively). In the resistant treated compartment, there was a large proportion of T-cells (37.2%) indicating that treatment could still participate in the recruitment of T-cells but might not cause an immune response. Epithelial cells, macrophages, and fibroblasts also composed 16.4%, 25.9% and 13.7% of resistant treated compartment suggesting their involvement in resistance development. Platinum-sensitivity and immune cell involvement in different stages were also evaluated (**Supplemental Figure S8A-B**). Regardless of treatment, samples from early stages tumors (I, II) were dominated by sensitive cells (87% and 81% sensitive cells, respectively) while late stages (III, IV) were dominated by resistant cells (70% and 55% resistant cells, respectively), highlighting increased resistance in later stages of disease as seen in the clinic. Across any stage, platinum-sensitivity was dominated by increased proportion of T-cells, while platinum-resistance was dominated by epithelial cells, macrophages, and fibroblasts.

Given the prevalence of macrophages in cells identified as platinum-resistant (particularly M2 macrophages), the effects of M2 macrophages on platinum-sensitive and platinum-resistant ovarian cancer cells growth dynamics could be quantified and was tested *in vitro* (Figure 3D). Naïve Tyk-nu and cisplatin-resistant Tyk-nu cp.r ovarian cancer cells were seeded in 1:1 co-cultures in triplicate in a 24-well plate. Cells were then exposed to standard media or TAM conditioned media for 14 days. Wells were imaged daily, and cell counts were abstracted using QuPath. Logistic growth rates and carrying capacities were estimated for each cell type and condition (**Supplemental Figure S9A**). Tyk-nu sensitive cells showed similar growth rates under standard and M2 TAM conditioned media (1.09-fold change difference), while Tyk-nu cp.r resistant cells under M2 TAM media showed a 1.46-fold change reduction in growth rate compared to those under standard media. While resistant cells grew slower than sensitive cells under both media conditions, their carrying capacity was 2.11-folds higher than sensitive cells under M2 TAM conditioned media. Thus, when evaluating the final population composition at day 14 under both media conditions, standard and M2 TAM conditioned media showed the same total population but at day 14 there was a much higher proportion of resistant cells (60.7%) when cultured with M2 TAM conditioned media compared to standard media (45.2%) (**Supplemental Figure S9B**). This suggested that while sensitive cells had a faster growth rate than resistant cells under both medias, M2 TAM conditioned media plateaued sensitive cells to carrying capacity around day 7, while resistant cells continue to proliferate and dominate in the population.

### Two distinct resistant signatures were identified suggesting differences between resistant primary and metastatic cells (580 words)

**Figure 4.**
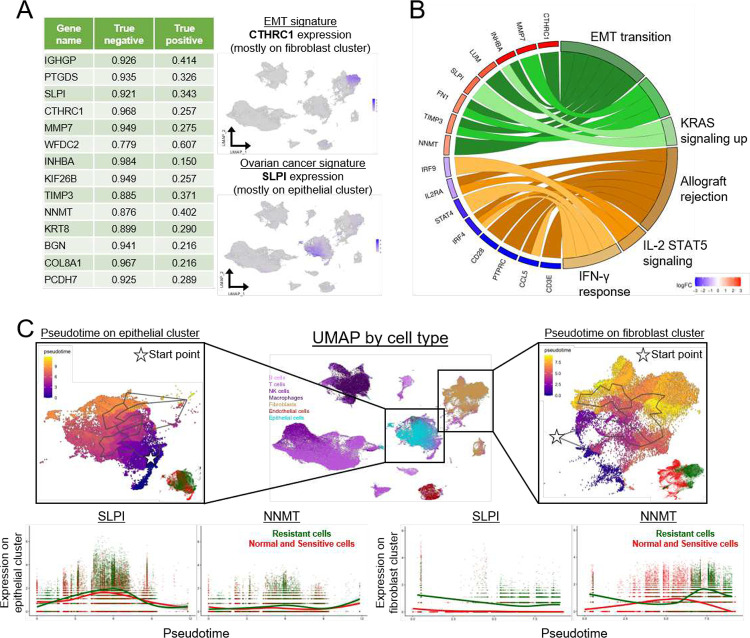
Resistant epithelial and fibroblast cell populations showed distinct gene signatures, which could be related to canonical or invasive ovarian cancer phenotypes. **A**. Top 15^th^ percentile of COMET genes upregulated in resistant versus all other cells (normal and sensitive cells) from single-cell RNA-seq differential gene expression. Feature plot of characteristic gene expression for EMT (CTHRC1) had highest expression in the fibroblast cluster, whereas expression of a canonical gene of ovarian cancer (SLPI) had highest expression in the epithelial cluster. **B**. Upregulated and downregulated Hallmark pathways in resistant cells compared to all other cells. EMT transition and KRAS signaling were upregulated in resistant populations, while sensitive cells had upregulated immune response pathways such the IL-2 STAT5 pathways and IFN-γ response. **C**. Monocle pseudotime analysis followed the trajectory from platinum-sensitive phenotype towards a resistant phenotype in the epithelial and fibroblast clusters. On the left, the pseudotime on the epithelial trajectory traced pseudotime from sensitive to resistant cells. The expression of a characteristic ovarian cancer gene, SLPI, was highly expressed across the entire pseudotime with highest expression in time points of higher density resistant cells. A characteristic EMT gene, NNMT, showed low expression in both sensitive and resistant cells across the entire pseudotime. On the right, the pseudotime on the fibroblast trajectory traced pseudotime from sensitive to resistant cells. SLPI showed low expression across the entire pseudotime, while NNMT, characteristic of fibroblasts, was highly expressed on resistant cells across the entire pseudotime with the highest expression at the end of the pseudotime where density of resistant cell was highest.

COMET was used to refine the differential expressed gene list to identify a putative gene signature to identify resistant cells (Figure 4A). Using the significantly upregulated genes in resistant cells, COMET ranked the genes based on enrichment and expression. A total of 94 genes characteristic of resistance were identified with top genes having an average log2-fold change of 2.3, an average true positive rate of 32%, and an average true negative rate of 92% (**Supplemental Figure S10A and Supplemental Table S5**). The expression of these genes across 2D umaps was evaluated using both the binary and XL-mHG test outputs (**Supplemental Figure S10C**) and two distinct resistant populations were identified.

A resistant fibroblast-like population that showed an EMT signature characterized by the genes PTGDS, CTHRC1, INHBA, TIMP3, NNMT, and BGN, and resistant epithelial-like population that showed a canonical ovarian cancer signature characterized by the genes SLPI, MMP7, WFDC2 and KRT8. Additionally, the upregulated and downregulated significant genes in resistant cells against all other cells were used to identify relevant significant hallmark pathway driven by resistance (Figure 4B). The upregulated pathways identified were EMT transition, coagulation and KRAS signaling (adj. p-value < 0.0001), while the downregulated included allograft rejection, IL-2 STAT5 signaling and IFN-γ response (adj. p-value < 0.005). While sensitive tumors elicited an immune response through the IL-2 STAT5 and IFN-γ pathways, the EMT transition may have aided in the development of resistance. Similar upregulated and downregulated pathways were observed when comparing resistant cells against sensitive cells only (**Supplemental Figure S3B**).

To explore the dynamics of expression profiles across resistance development, pseudotime analysis was ran in the single-cell RNA-seq cohort using Monocle (Figure 4C). Pseudotime trajectories were analyzed distinctly for epithelial phenotype changes as well as fibroblast phenotype changes. The two cell populations were subset based on cluster assignment with cluster 2 being a mostly epithelial cluster (57% of all cells in cluster 2) and cluster 3 being a mostly fibroblast cluster (89% of all cells in cluster 3) (**Supplemental Figures S3D and E**). A non-resistant cell in each cluster was chosen as starting point to trace the RNA trajectory from more platinum-sensitive to platinum-resistant. Then using top genes identified from COMET, the expression profile from a representative gene from each signature (EMT vs ovarian cancer) were then plotted along the pseudotime. For the epithelial trajectory, the expression of SLPI (characteristic of the ovarian cancer signature) was highly expressed in resistant cells across the entire pseudotime, particularly in regions of high density of resistant cells. Meanwhile, the expression of NNMT (characteristic of EMT) was low in both sensitive and resistant phenotypes over pseudotime. The inverse trend was observed for these same genes across the fibroblast trajectory. The expression of SLPI was low over pseudotime for all cells, while the expression of NNMT was high in resistant cells, particularly at the end of the pseudotime were there was a higher density of resistant cells. These results suggest that there are at least two pathways to evolve platinum-resistant ovarian cancer cell populations. The change in the fibroblast population may be attributed to increased EMT signaling potentially leading to a more invasive phenotype, while epithelial cells continue to promote resistance from the tumor site.

**Figure 5.**
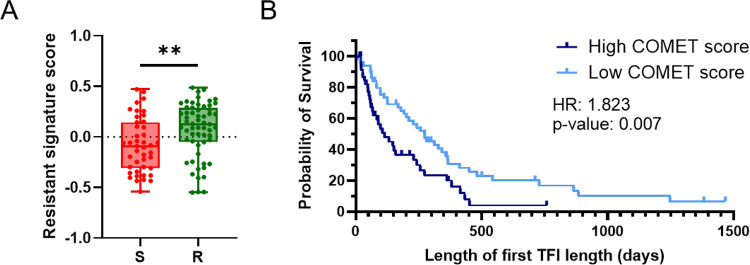
Validation of resistant gene signature identified with COMET in the bulk TCGA-OV dataset. **A**. Box and whiskers plot shows higher resistant signature scores in the TCGA-OV patients labeled resistant compared to sensitive. **B**. Survival plot grouping patients in high and low groups (see Methods) indicates that patients with lower resistant scores had significantly longer first TFIs compared to those with higher resistant scores (HR: 1.823, Cox p-value: 0.007). Unpaired t tests were used to compared data with two groups. ** p-value < 0.01

Even though there were no differences in cell type markers between sensitive and resistant samples from the TCGA-OV cohort, the entire resistant signature identified with COMET was scored for each patient in the TCGA-OV cohort. Patients classified as resistant had an absolute change in resistant signature score by 0.153 compared to patients classified as sensitive (Figure 5A). Using the first treatment-free interval (TFI) length as the time-to-event variable, patients with high COMET scores were associated with lower survival and a median TFI of 115 days which classify them as resistant (HR: 1.823, Cox p-value: 0.007) (Figure 5B). In contrast, patients with low COMET scores were associated with higher survival and a median TFI of 273 days which would classify them as sensitive. Additionally, the expression of genes such as SLPI (p-value: 0.0008), MMP7 (p-value: 0.0272), WFDC2 (p-value: 0.0142) and NNMT (p-value: 0.0012) was up to 1.2-fold higher in resistant samples compared to sensitive samples (**Supplemental Figure S10D**).

## DISCUSSION

Ovarian cancer remains the deadliest gynecological cancer. While at early stages, surgery and platinum-based chemotherapy can achieve a 5-year survival rate of 91.7%, however, most cases are diagnosed at advanced stages once the disease has spread to the peritoneum and other organs(1,2). At those stages, platinum-based chemotherapy and targeted therapies may result in initial remission, but recurrence may occur in up to 80% of patients (47,48). Given that novel targeted therapies developed in recent decades have failed to improve the prognosis of ovarian cancer, there has been a shift to focus treatment strategies to disrupt the tumor microenvironment (TME). In ovarian cancer, the TME is a heterogeneous complex structure composed of several stromal and immune cell types that might play a role in treatment response and resistance. Here, we aim to study the TME using different technologies and a variety of samples ranging from normal ovarian tissues and ovarian cancer tissues at different stages, treatment status, and platinum-sensitivities (Figure 1).

To unravel the cellular composition difference between normal (non-cancerous) and cancerous ovarian tissue, cellular phenotypes were assessed through mIF, bulk RNA-seq, and scRNA-seq (Figure 2 **and Supplemental Figures S1-S3**). Across the three modalities, it was observed that macrophages (CD163+), B-cells (CD20/MS4A1+), and T-cells (CD8A+) cells were highly expressed in cancer samples compared to normal. Fibroblasts (α-SMA+/ACTA2+) were depleted in cancer samples, which has also been shown by other studies (49,50). While ACTA2 is expressed in many CAF populations, normal stromal and myofibroblast populations also express this gene. Studies have found that, in addition to ACTA2, other markers need to be co-expressed to lead to malignancy (51–53). Overall, in the ovarian landscape, tumor-associated immune cells were being recruited to the tumor site, while stromal components were depleted potentially allowing for the re-shaping of the tumor site. To compare the agreement between the technologies, we used the Bland-Altman method and pathway analysis (**Supplemental Figure S4 and S5**). Results indicated all technologies can be used interchangeably to capture CD163+, CD20+ and α-SMA+ cell proportions across non-cancerous and cancerous ovarian samples. Additionally, both bulk RNA- and scRNA-seq showed upregulation of immune and inflammatory pathways such as TNF-α signaling via NF-kB, the inflammatory response, IL-2 STAT5 pathway and interferon alpha and gamma response (IFN-α and IFN-γ). These pathways promote an inflammatory tumor niche that contributes to ovarian cancer invasion, proliferation, apoptosis evasion and angiogenesis as well as the recruitment of M2 macrophages and T cells directly related to the results seen on Figure 2 (54–57). Interestingly, the most significant downregulated pathway in cancer compared to normal samples was the EMT pathway. In ovarian cancer, EMT is often a partial or transient state showing upregulation only in the resistant population (58,59). Taken together, ovarian cancer samples are characterized by the upregulation of inflammatory and immune responses that contribute to tumor progression through the presence of T cells and M2 macrophages; and the downregulation of extracellular structural programs contributing to loss of cellular adhesion points and the decrease of fibroblasts.

A subset of single-cell RNA-seq patient samples were clinically classified as platinum-sensitive or platinum resistant, which enabled a deeper characterization of the cell types and pathways responsible for driving platinum resistance (Figure 3A–C). Platinum-sensitive samples were enriched by T-cells and NK-cells, while resistant samples were enriched with macrophages, CAFs, and epithelial cells. This suggests that sensitive samples have an immune-infiltrated microenvironment that could lead to anti-tumor responses while the microenvironment of resistant samples has shifted towards an immuno-suppressive pro-tumor niche that is associated with cancer resistance (60). These compositional microenvironment patterns in platinum-sensitive versus platinum-resistant were also identified in treatment-naïve versus treated samples, suggesting that tumor resistance may be driven by macrophages, fibroblasts, and epithelial cells regardless of treatment exposure. This further complicates the ovarian cancer TME contributing to why immunotherapies targeting T-cell cytotoxicity have not yet been successful clinically, despite their high presence in both sensitive and resistant tumors (42,61,62).

Studies have shown that ovarian cancer cells are more invasive and chemo-resistant in the presence of M2 macrophages (63–65), however the difference in growth dynamics between sensitive and resistant cells has not yet been reported. To test the effects of M2 macrophages on the growth dynamics of platinum-sensitive and platinum-resistant ovarian cancer cells *in vitro*, co-cultures under standard and M2 tumor-associated macrophage (TAM) conditioned media were performed. Growth curves showed that while M2 TAM conditioned media resulted in a slower resistant cell growth rate compared to standard media, the carrying capacity was increased resulting in the dominance of the resistant cells (Figure 3D). While differences between cell type specific markers were able to be assessed in the single-cell RNA-seq dataset, analysis of these phenotype markers (CD163, CD20, CD8A, and α-SMA) on sensitive and resistant patients in a bulk RNA-seq dataset downloaded from TCGA revealed no significant differences. This indicates that additional transcriptomic resolution is needed to unravel these differences.

Differential gene expression identified resistant cells have an upregulation of EMT, coagulation and KRAS signaling programs, while downregulating allograft rejection, IL-2 STAT5 signaling and IFN-γ response pathways (Figure 4). These results were opposite of trends between cancer versus normal samples suggesting that resistant cells have distinct gene expression and pathway patterns. Resistant cells can acquire a mesenchymal-like state allowing them to invade and metastasize to different locations, while evading the effects of chemotherapeutics. This has been shown in resistant ovarian cancer cells *in vitro* (66,67) and *in vivo* including that the blockage of EMT can overcome chemoresistance (68). In addition, KRAS mutations in ovarian cancer are associated with poor outcomes and chemoresistance (69). In contrast, the downregulation of immune pathways such as IL-2 STAT5 pathway and IFN-γ response suggests attenuation of immune activation consistent with the decrease in T-cell population in resistant samples compared to sensitive (70).

Further ranking of genes associated with platinum-resistant cells were used to generate two distinct signatures of platinum-resistance. Genes such as SLPI, MMP7, WFDC2, and KRT8 were only expressed in the epithelial cells and are known to be characteristic of ovarian cancer cells. Particularly, the overexpression of SLPI and WFDC2 have been shown to promote chemoresistance and tumor growth in ovarian cancer (46,71–73). Additionally, high expression of MMP7 and KRT8 have been shown to mediate cell proliferation and survival in cancers including ovarian, pancreatic, and breast (74–76). These genes form part of an epithelial-like ovarian cancer signature that promotes chemoresistance.

Genes such as PTGDS, CTHRC1, INHBA, TIMP3, KIF26B and NNMT which were all highly expressed only in the fibroblast cluster. These genes are characteristic of a mesenchymal and stromal invasion resistance signatures, suggesting that the fibroblast-like population acquires a more invasive phenotype. Studies have shown that high expression of CTHRC1, INHBA, TIMP3, and KIF26B is associated with aggressive CAFs in the ovarian and pancreatic tumor microenvironment leading to an immunosuppressive TME, the promotion of EMT, chemo-resistance, and poor prognosis (52,77–79). NNMT has also been studied in the context of different types of ovarian cancer and shown that high expression is associated with CAFs in the TME as well as resistance, while inhibition of NNMT can restore antitumor immune responses (80–83).

These two distinct resistant signatures in ovarian cancer where further explored through RNA trajectory analysis using pseudotime, where EMT resistant genes were only expressed in fibroblast-like resistant cells and the ovarian cancer canonical resistant genes were only expressed in epithelial-like resistant cells. While the identification of resistant gene signature was performed on the single-cell RNA-seq dataset, the bulk RNA-seq TCGA-OV patients were scored based on the expression of the 94 genes of the signature (Figure 5). Patients with high resistant signature scores were predicted to have a significantly lower first TFI length with a median of 115 days which would classify then as resistant. While patients with a lower resistant signature score were predicted to have a much longer first TFI length with a media of 273 days which would classify them as sensitive. This score could be used as a predictive tool for resistance where high scoring patients should be deviated from the standard of care towards treatment schedules that could delay the onsets of resistance.

## CONCLUSIONS

This study characterized the ovarian cancer TME landscape using three different technologies mIF, bulk and single-cell RNA-seq. Overall, we found that ovarian cancer is associated with increased immune and inflammatory pathways along with high infiltration of macrophages and T cells relative to normal tissue, suggesting a pro-tumor inflammatory environment that promotes invasion and proliferation. In contrast, the downregulation of structural pathways and depletion of fibroblasts in cancer samples indicates substantial remodeling of the tumor niche. In terms of treatment resistance, our analysis suggests that both macrophages and fibroblasts contribute to treatment-evasion, with fibroblasts showing increased expression of EMT-related genes suggesting phenotypic plasticity towards a more invasive phenotype. We also identified a novel resistant gene signature from the scRNA-seq cohort and validated it in a bulk RNA-seq cohort, supporting its potential use a predictive biomarker for resistance. Together, these findings highlight the importance of tumor microenvironment, particularly macrophages and fibroblasts, in ovarian cancer progression and resistance, and establish a resistant signature for aiding future treatment decisions, biomarker discovery and therapeutic targeting.

## Supplementary Material

Supplementary Files

This is a list of supplementary files associated with this preprint. Click to download.

- DelPinoHerreraetalOvCaTMESupplTables2026.xlsx
- DelPinoHerreraetalOvCaTMESupplMethodsFigs2026.pdf

## Data Availability

The datasets generated and analyzed during the current study are available in a GitHub repository at: https://github.com/mcfefa/OvCa-TME and a reproducible run on CodeOcean Capsule at: https://codeocean.com/capsule/2396450/tree

## LIST OF ABBREVIATIONS

TME: tumor microenvironment
mIF: multiplex immunohistochemistry
Bulk RNA-seq: bulk RNA-sequencing
scRNA-seq: single-cell RNA-sequencing
HGSOC: high-grade serous ovarian cancer
PARP: poly (ADP-ribose) polymerase
CAF: cancer-associated fibroblast
TAM: tumor-associated macrophage
ROI: region of interest
FFPE: formalin-fixed paraffin-embedded
CTSI: clinical and translational science institute
BioR: biorepository
GEO: gene expression omnibus
TCGA: the cancer genome atlas
TFI: treatment-free interval
EMT: epithelial to mesenchymal transition
